# Chronic Kidney Disease Induced Intestinal Mucosal Barrier Damage Associated with Intestinal Oxidative Stress Injury

**DOI:** 10.1155/2016/6720575

**Published:** 2016-07-14

**Authors:** Chao Yu, Zhen Wang, Shanjun Tan, Qiang Wang, Chunyu Zhou, Xin Kang, Shuang Zhao, Shuai Liu, Huijun Fu, Zhen Yu, Ai Peng

**Affiliations:** ^1^Department of Nephrology & Rheumatology, Shanghai Tenth People's Hospital, Tongji University School of Medicine, Shanghai 200072, China; ^2^Department of General Surgery, Zhongshan Hospital, Fudan University, Shanghai 200032, China; ^3^Department of Pathology, Shanghai Tenth People's Hospital, Tongji University School of Medicine, Shanghai 200072, China; ^4^Department of General Surgery, Shanghai Tenth People's Hospital, Tongji University School of Medicine, Shanghai 200072, China

## Abstract

*Background.* To investigate whether intestinal mucosal barrier was damaged or not in chronic kidney disease progression and the status of oxidative stress.* Methods.* Rats were randomized into two groups: a control group and a uremia group. The uremia rat model was induced by 5/6 kidney resection. In postoperative weeks (POW) 4, 6, 8, and 10, eight rats were randomly selected from each group to prepare samples for assessing systemic inflammation, intestinal mucosal barrier changes, and the status of intestinal oxidative stress.* Results.* The uremia group presented an increase trend over time in the serum tumor necrosis factor-alpha, interleukin-6 (IL-6) and IL-10, serum D-lactate and diamine oxidase, and intestinal permeability, and these biomarkers were significantly higher than those in control group in POW 8 and/or 10. Chiu's scores in uremia group were also increased over time, especially in POW 8 and 10. Furthermore, the intestinal malondialdehyde, superoxide dismutase, and glutathione peroxidase levels were significantly higher in uremia group when compared with those in control group in POW 8 and/or 10.* Conclusions.* The advanced chronic kidney disease could induce intestinal mucosal barrier damage and further lead to systemic inflammation. The underlying mechanism may be associated with the intestinal oxidative stress injury.

## 1. Introduction

Chronic kidney disease (CKD) is a common chronic disease and often progressively develops to be the end-stage kidney disease [1]. The average survival period is 4.25 years in uremia patients, and the 1-year, 3-year, and 5-year survival rates are 88%, 68%, and 46%, respectively [2]. It is reported that CKD was ranked the 27th cause for global deaths in 1990, but it rose to be the 18th in 2010, and the number is also increasing in the past several years [3]. In clinical practice, patients who suffer from CKD need more medical and social care, increasing the medical costs and affecting the quality of life [4–7]. Therefore, how to delay the disease progression and exploring effective treatments to reduce complications are popular pursuits in modern nephrology.

Many studies have shown that the infectious complications are one of the main causes for the CKD deterioration [8–11]. For example, the mortality may increase dramatically in uremia patients with pulmonary infections, and infectious diseases may also lead to additional damage to the kidneys and other organs or tissues [12, 13]. However, it is unclear which is the originating site of the infection. As we know, gut is the largest community of bacteria, and the microorganisms are much more than the cells in human body [14]. In the normal condition, these microorganisms are located in the intestinal tract by the integrity of gut barrier including mechanical barrier, biological barrier, chemical barrier, and immunologic barrier. However, in the pathological condition, damage to the intestinal mucosal barrier will result in intestinal bacterial and endotoxin translocation and further contribute to local and systemic inflammation [15–17]. The loss of intestinal mucosal barrier has been considered as one of the most important causes for the infection in various diseases [18, 19]. Furthermore, in clinical practice, patients with CKD often show local and systemic inflammatory syndrome [20–22]. However, up to now, it is still unknown whether the intestinal mucosal barrier is damaged or not in the progression of CKD.

In addition, as we know, there are many uremic toxins in the body of patients with CKD, especially the intestine [23]. These uremic toxins could cause oxidative stress injury to the organs or tissues when the increased free radical oxygen species overwhelm the body's normal ability to eliminate them [24, 25]. Malondialdehyde (MDA) is an end-product of lipid peroxidation in the oxidative stress process, and superoxide dismutase (SOD), catalase (CAT), and glutathione peroxidase (GSH-PX) are the main antioxidant enzymes protecting the body from oxidative stress injury. These biomarkers are commonly used to evaluate oxidative stress in many researches [26–28]. However, up to now, it is also unknown whether the oxidative stress injury is involved in the intestinal mucosal barrier damage in the progression of CKD.

In the present study, therefore, we aim to investigate whether the intestinal mucosal barrier was damaged or not in the progression of CKD and the status of oxidative stress responsible for the underlying mechanism.

## 2. Materials and Methods

### 2.1. Animals and Ethics Statement

Healthy adult male Sprague-Dawley rats (weighing 170 to 200 g) were obtained from Shanghai Tenth People's Hospital, Shanghai, China. The rats were housed in our laboratory in a temperature- and humidity-controlled environment. All rats were housed under a normal 12-hour light/dark cycle and with access to food and water ad libitum. This animal use and care protocol and experimental procedures were reviewed and approved by the Institutional Animal Care and Use Committee of Shanghai Tenth People's Hospital. The experiments were also performed according to the National Institutes of Health Guidelines on the use of laboratory animals.

### 2.2. Experimental Protocol and Sample Collection

After an adaptation period for one week, sixty-four rats were randomly divided into two groups: a control group and a uremia group, with 32 in each. The rat model of uremia was induced by 5/6 kidney resection as described in the previous study [29]. Briefly, after full anesthesia with subcutaneous injection of 2% pentobarbital sodium (3.5 mL/kg), the rats in the uremia group underwent laparotomy with a 2 cm dorsal incision, after which the 2/3 of the left kidney was removed, and seven days later, the whole right kidney was removed. The control group went through the same two procedures but without any kidney resection. The surgical procedures were performed in an aseptic environment with controlled temperature and humidity.

In postoperative weeks 4, 6, 8, and 10, eight rats were randomly selected from each group, and samples of blood and intestinal tissues were obtained after animals were fully anesthetized. Blood samples were obtained from the inferior vena cava, and the serum was prepared by centrifugation at a speed of 1500 rpm for 15 min at 4°C, and then the serum was stored at −80°C for analyses of cytokines and D-lactate (D-LA) levels and diamine oxidase (DAO) activity. The terminal ileum was collected for analyses of intestinal permeability, oxidant and antioxidant levels, and histopathology. Of note, the selection of terminal ileum as the investigation site was based on the previous studies [30–32]. These studies have indicated that the terminal ileum is the major site for observation of damage to intestinal mucosal barrier in various diseases. It is suggested that terminal ileum is the most sensitive section of the intestinal tract [33].

### 2.3. Serum Tumor Necrosis Factor-Alpha (TNF-*α*), Interleukin-6 (IL-6), and IL-10 Determination

The levels of cytokines tumor TNF-*α*, IL-6, and IL-10 in the serum were determined using an ELISA kit for rats (R&D Systems, Minneapolis, MN, USA) according to the manufacturer's instructions. The values were expressed as pg/mL in the serum.

### 2.4. Serum D-LA and DAO Determination

The D-LA levels and DAO activity in the serum were determined using ELISA kits for rats (for D-LA, R&D Systems, Minneapolis, MN, USA; for DAO, Nanjing Jiancheng Biocompany, Nanjing, China) in accordance with the manufacturer's instructions. The D-LA level was expressed as mmol/L in the serum, and the DAO activity was expressed as U/mL in the serum.

### 2.5. Intestinal Permeability Determination

The intestinal permeability was determined by measurement of intestinal clearance of fluorescein-isothiocyanate dextran (FD4) as reported in our previous studies [15, 30, 34]. Briefly, a terminal ileum segment with 8 cm length was collected, and the mucosa was gently everted. At one end, the gut segment was ligated, and from the other end, a gut sac was prepared by injecting 1.0 mL of Krebs-Henseleit bicarbonate buffer. The filled sac was then incubated in a solution containing 0.5 mg/mL of FD4 (average molecular weight: 4000) at controlled temperature with 37°C. The bathing solution was also aerated by gently bubbling with a gas mixture containing 5% CO_2_ and 95% O_2_. Thirty minutes later, the value of the mucosal surface area (*A*) was measured. The fluorescence of the solution was then measured by a fluorescence spectrophotometer and the intestinal clearance of FD4 was calculated according to the following formula:

(1) $$\begin{array}{c} C=\frac{{\left[FD4\right]}_{ser}\times 1\;mL}{{\left[FD4\right]}_{muc}\times A\times 30\;min}\;A=\pi LD. \end{array}$$

In this formula, *C* is the mucosal-to-serosal clearance of FD4 in *μ*L·min^−1^·cm^−2^, [FD4]_ser_ is the FD4 concentration in the serosal fluid aspirated from the gut sac at the end of the 30 min period, [FD4]_muc_ is the FD4 concentration in the mucosal fluid aspirated from the gut sac at the beginning of the 30 min period, *L* is the length of the gut sac, and *D* is the diameter of the gut sac.

### 2.6. Intestinal MDA, SOD, CAT, and GSH-PX Determination

The terminal ileum was homogenized and centrifuged at a speed of 4,000 rpm for 15 min at 4°C, and then the supernatant was obtained. The level of MDA and the activity of SOD, CAT, and GSH-PX in the supernatants were determined using the commercial analysis kits (Nanjing Jiancheng Biocompany, Nanjing, China) in accordance with the manufacturer's instructions. The MDA level was expressed as nmol/mg in the tissue, and the SOD, CAT, and GSH-PX activity were expressed as U/mg in the tissue.

### 2.7. Histological Observation

The terminal ileum was fixed with 10% buffered formaldehyde and embedded in paraffin. Slices of 4 *μ*m thick were made, stained with hematoxylin and eosin (H&E), and then observed by two pathologists blinded to this study design with light microscopy. The infiltration of inflammatory cells such as neutrophils was employed to assess the intestinal inflammation in the intestinal mucosa and submucosa [30]. The degree of intestinal mucosal injury was quantitatively assessed using Chiu's scoring system reported by Chiu et al. [35].

### 2.8. Statistical Analysis

Data were expressed as the mean ± SD. Statistical analyses were made using SPSS 17.0 software (SPSS Inc., Chicago, USA). Data received homogeneity test first for variance and were then analyzed using Student's *t*-test, except Chiu's scores using one-way analysis of variance followed by the Least Significant Difference test for multigroup comparisons. Differences were considered statistically significant when  *P* < 0.05.

## 3. Results

### 3.1. General Observations

**Figure 1:**
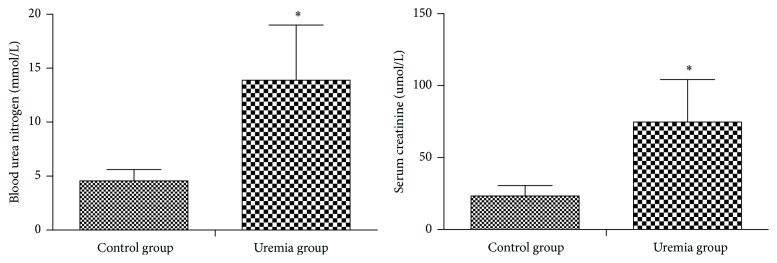
Serum levels of blood urea nitrogen and creatinine in each group in postoperative week 4. Data are expressed as mean ± SD. ^*∗*^ *P* < 0.05, versus control group.

All rats survived in the entire experiments. Four weeks after operation, a pronounced rat model of uremia, as indicated by the increase in serum creatinine concentrations and blood urea nitrogen (Figure 1), was induced successfully. Compared with the control rats, the food intake and locomotor of the rats in uremia group decreased evidently.

### 3.2. Serum Levels of Cytokines TNF-*α*, IL-6, and IL-10

**Figure 2:**
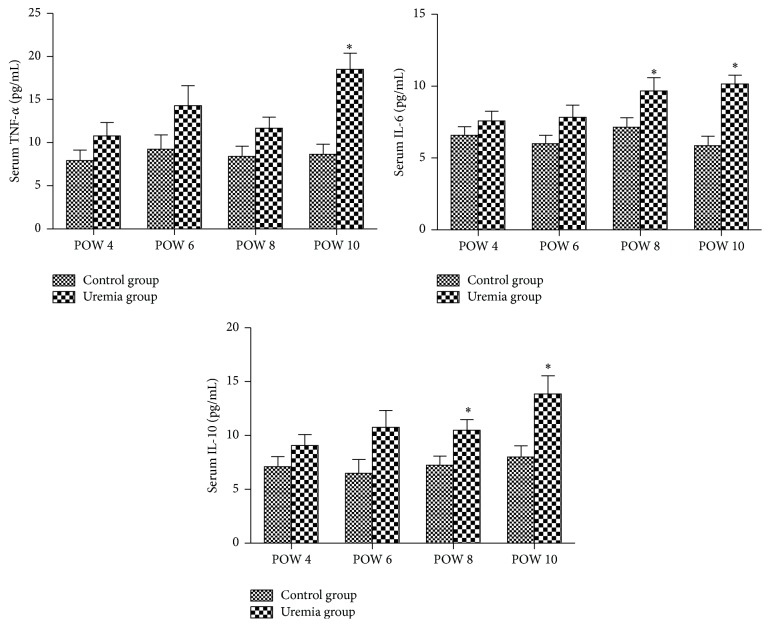
Serum levels of inflammatory cytokines TNF-*α*, IL-6, and IL-10 in each group. POW: postoperative week. Data are expressed as mean ± SD. ^*∗*^ *P* < 0.05, versus control group at the same time point.

The results are shown in Figure 2. The serum levels of TNF-*α*, IL-6, and IL-10 in the uremia group presented an increased trend over time, and these cytokine levels were higher than those in the control group at all the investigated time points. For the proinflammatory cytokine TNF-*α*, there was a significant difference between the uremia group and the control group in postoperative week 10 (*P* < 0.05). For the proinflammatory cytokine IL-6, the difference between these two groups reached significance in postoperative weeks 8 and 10 (*P* < 0.05). In addition, on both postoperative weeks 8 and 10, the serum level of the anti-inflammatory cytokine IL-10 of the uremia group was also significantly increased when compared with that of the control group (*P* < 0.05).

### 3.3. Serum D-LA Levels and DAO Activity

**Figure 3:**
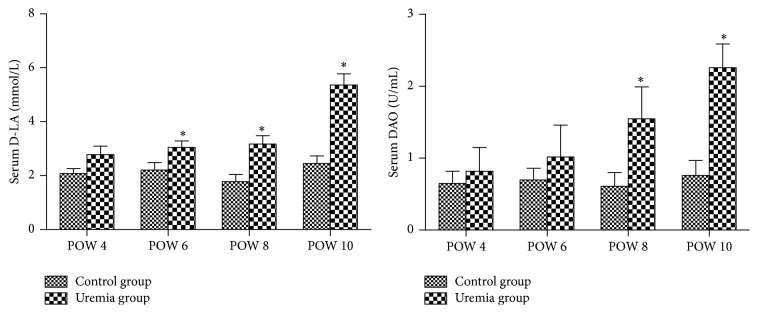
Serum LA levels and DAO activity in each group. POW: postoperative week. Data are expressed as mean ± SD. ^*∗*^ *P* < 0.05, versus control group at the same time point.

The results are shown in Figure 3. The serum D-LA levels and DAO activity in the uremia group presented an increased trend over time, and these biomarker levels were higher than those in the control group at all the investigated time points. For the D-LA, there was a significant difference between the uremia group and the control group in postoperative weeks 6, 8, and 10 (*P* < 0.05). For the DAO, the difference between these two groups reached significance in postoperative weeks 8 and 10 (*P* < 0.05).

### 3.4. Intestinal Permeability

**Figure 4:**
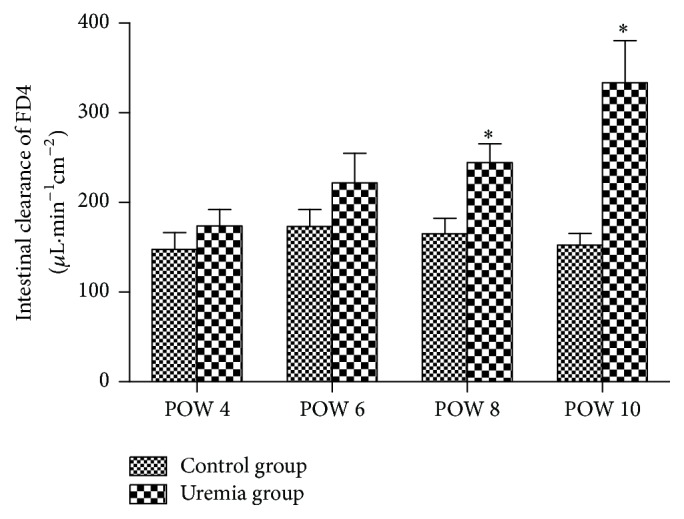
Intestinal clearance of FD4 in each group. POW: postoperative week. Data are expressed as mean ± SD. ^*∗*^ *P* < 0.05, versus control group at the same time point.

The results are shown in Figure 4. The intestinal clearance of FD4 in the uremia group presented an increased trend over time, and these biomarker levels were higher than those in the control group at all the investigated time points. In postoperative weeks 8 and 10, the intestinal clearance of FD4 of the uremia group was significantly increased when compared with that of the control group (*P* < 0.05).

### 3.5. Histopathology

**Figure 5:**
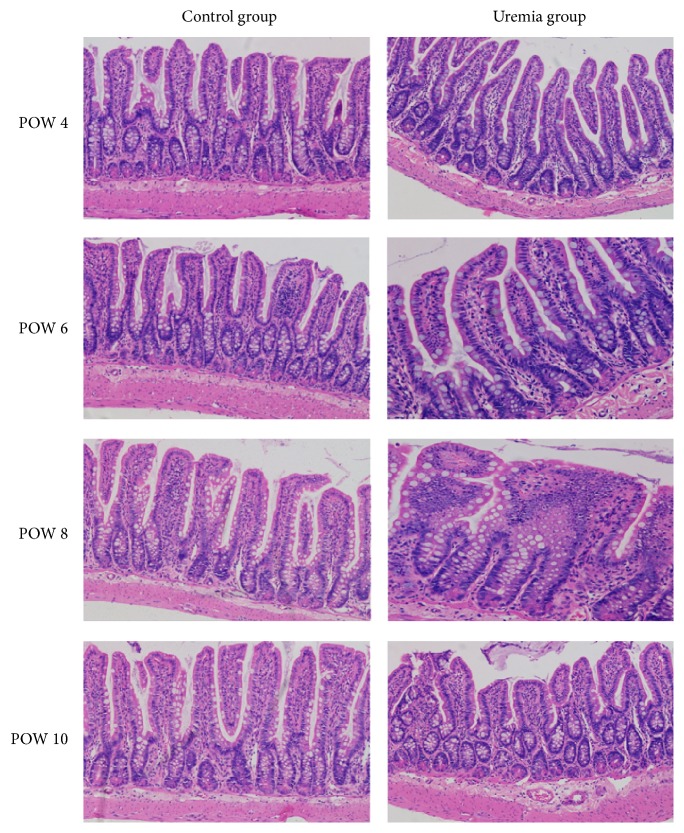
Representative microscopic changes of the intestinal tissue stained with H&E using light microscopy (100x) in each group. POW: postoperative week. There were time-dependent edema and inflammation in the intestinal mucosa and submucosa in uremia group when compared with those in the control group, especially in postoperative weeks 8 and 10.

**Figure 6:**
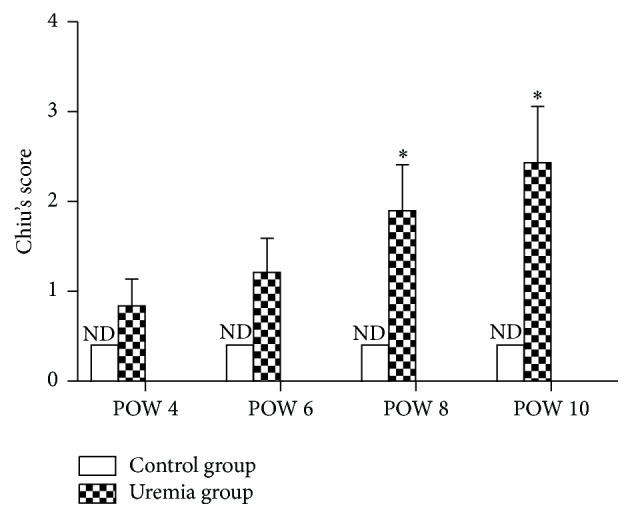
Chiu's score in each group. ND: not detectable; POW: postoperative week. Data are expressed as the mean ± SD. ^*∗*^ *P* < 0.05, versus uremia group in POW 4.

The results are shown in Figure 5. No obvious structural injury was observed in the intestinal tissue between the uremia group and the control group at all the investigated time points. However, edema and inflammatory cells were observed in the intestinal mucosa and submucosa in uremia group when compared with those in the control group. In addition, as shown in Figure 6, the uremia group presented an increased trend in Chiu's score over time, and the scores in postoperative weeks 8 and 10 were higher than those in postoperative week 4 (*P* < 0.05).

### 3.6. Intestinal Levels of MDA, SOD, CAT, and GSH-PX

**Figure 7:**
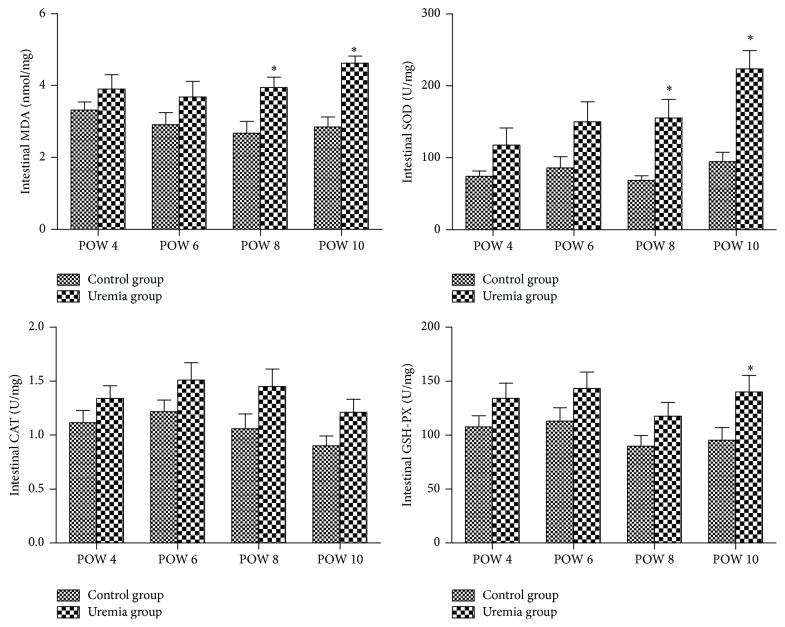
The intestinal levels of MDA, SOD, CAT, and GSH-PX in each group. POW: postoperative week. Data are expressed as mean ± SD. ^*∗*^ *P* < 0.05, versus control group at the same time point.

The results are shown in Figure 7. The intestinal levels of MDA, SOD, CAT, and GSH-PX in the uremia group presented an increased trend over time, and these biomarker levels were higher than those in the control group at all the investigated time points. For the MDA and SOD, the differences between these in the uremia group and the control group reached significance in postoperative weeks 8 and 10 (*P* < 0.05). In addition, in postoperative week 10, the intestinal levels of GSH-PX of the uremia group were significantly increased when compared with those of the control group (*P* < 0.05). However, the intestinal levels of CAT of the uremia group were not significantly increased when compared with those of the control group at all the investigated time points (*P* > 0.05).

## 4. Discussion

In the present study, we investigated whether the intestinal mucosal barrier was damaged or not in the progression of CKD and the status of oxidative stress responsible for the underlying mechanism. Our results showed that the uremia group presented an increased trend over time in the serum cytokines TNF-*α*, IL-6, and IL-10, the serum D-LA and DAO, and the intestinal permeability, and these biomarkers were higher than those in the control group at all the investigated time points, especially in postoperative week 8 and/or 10. Meanwhile, histopathological findings showed that edema and inflammatory cells were observed in the intestinal mucosa and submucosa in the uremia group, although there was no substantial injury in this group. However, the uremia group presented an increased trend over time in Chiu's scores assessed for measuring intestinal mucosal injury, and the scores in postoperative weeks 8 and 10 were higher than those in postoperative week 4. In addition, the increases in the intestinal levels of MDA, SOD, CAT, and GSH-PX were time-dependent in the uremia group, and the levels of MDA, SOD, and GSH-PX were significantly increased when compared with the control group in postoperative week 8 and/or 10. These results revealed that the advanced CKD could induce intestinal mucosal barrier damage and further lead to systemic inflammation, and the underlying mechanism may be associated with the oxidative stress injury in the intestine.

CKD is a common chronic disease and often progressively develops to be the end-stage kidney disease [1]. In clinical practice, patients with CKD often suffer from various complications [8, 12], and, therefore, they may need more medical and social care, increasing the medical costs and affecting the quality of life [4–6]. It is a main target to reduce complications so as to delay the disease progression. However, there are limited studies linking to the cause for the complications followed by CKD. As we know, various infectious diseases are one of the main complications of CKD, and they could lead to CKD deterioration [8–10]. For example, the mortality may increase dramatically in uremia patients with pulmonary infections, and infectious diseases may also lead to additional damage to the kidneys and other organs or tissues [12]. However, the originating site of these infections after CKD progression is still unknown.

As we know, gut is the largest community of bacteria, and the microorganisms are much more than the cells in the human body [14]. In the normal condition, these microorganisms' colonization of the gut is confined to the gastrointestinal tract by the integrity of gut barrier including mechanical barrier, biological barrier, chemical barrier, and immunologic barrier. However, in the pathological condition, damage to the intestinal mucosal barrier will result in intestinal bacterial and endotoxin translocation and further contribute to local and systemic inflammation [15–17]. The loss of intestinal mucosal barrier has been considered as one of the most important causes for the infection in various diseases [18, 19]. Furthermore, in clinical practice, patients with CKD often show local and systemic inflammatory syndrome [20–22]. In the present study, we also demonstrated that the uremia group presented an increased trend over time in the serum cytokines TNF-*α*, IL-6, and IL-10, and these cytokines were higher than those in the control group at all the investigated time points. The underlying mechanism for these systemic inflammations may be the results from the damage of intestinal mucosal barrier [15, 36]. Therefore, in order to further elucidate the mechanism of the systemic inflammation followed by CKD, it is important to determine the change of intestinal mucosal barrier in the status of CKD.

It is a challenging task to comprehensively assess the function of the intestinal mucosal barrier [30], but the intestinal permeability is often employed to indirectly determine the barrier function. For example, only tiny amounts of D-LA (the metabolic product of bacteria), DAO (an intracellular enzyme confined mostly in the intestinal villus cells), and FD4 (a relatively large molecule) can be detected in the serum under normal conditions, while increased concentrations will be detected when the intestinal mucosal barrier is damaged [15, 30, 33, 37–39]. Therefore, serum D-LA levels and DAO activity as well as the intestinal clearance of FD4 have all been often considered as sensitive biomarkers for assessing the intestinal mucosal barrier function. As expected, the serum D-LA and DAO, as well as the intestinal clearance of FD4, were elevated in the uremia group, especially in postoperative week 8 and/or 10. Meanwhile, histopathological findings showed that edema and inflammatory cells were observed in the intestinal mucosa and submucosa in the uremia group. Accordingly, increased Chiu's scores were recorded in the uremia group, especially in postoperative weeks 8 and 10. On these grounds, these results indicated that the intestinal mucosal barrier was damaged in the progression of CKD, and method to ameliorate intestinal mucosal barrier damage may be an effective strategy to reduce the infectious complications when patients suffer from CKD [40].

In addition, when we designed the present experiment, we were not sure which factor was responsible for the intestinal mucosal barrier damage following CKD. As we know, there are many uremic toxins in the patients body with CKD, especially the intestine [23]. These uremic toxins could cause oxidative stress injury to the organs or tissues when the increased free radical oxygen species overwhelm the body's normal ability to eliminate them [24, 25]. In previous studies, there were many tests to analyze oxidative stress such as detecting reactive oxygen species-derived products, but some biomarkers including MDA, SOD, CAT, and GSH-PX were mostly used, which were also employed in our previous studies [26, 27]. In the present study, we found that the level of intestinal MDA was gradually increased in the CKD progression when compared with the control group. As an end-product of lipid peroxidation, this MDA increase may be the result of oxidative stress injury followed by CKD progression. SOD, CAT, and GSH-PX are the main antioxidant enzymes, which could protect the body from oxidative stress injury. In the present study, to our surprise, the levels of intestinal SOD, CAT, and GSH-PX were also increased after CKD progression. The underlying mechanism for these antioxidant enzymes increases was not investigated in the present study; but we speculate that there may be a feedback or compensation mechanism for these increases to protect the target organ from oxidative stress injury in CKD progression [41, 42]. Increases in both the oxidative marker MDA and the antioxidant enzymes (SOD, CAT, and GSH-PX) levels reveal the increased oxidative stress response following CKD progression [24, 43]. In addition, it is reported that Nrf2-ARE pathway genes or others were originally identified as the molecular signature to correspond to a redox unbalance of cellular systems in stress conditions [44, 45]. Nevertheless, the further study emphasizing these genes and other pathways regarding mitochondrial oxidative stress mediated apoptosis is underway to explore the exact mechanism of the intestinal mucosal barrier damage after CKD progression.

In conclusion, our results suggest that the advanced CKD could induce intestinal mucosal barrier damage and further lead to systemic inflammation, and the underlying mechanism may be associated with the oxidative stress injury in the intestine. Therefore, preservation of intestinal mucosal barrier function through effective methods may be considered as a potential target for therapies aimed at preventing infectious complications after CKD progression.
